# Intramuscular Exposure of *Macaca fascicularis* to Low Doses of Low Passage- or Cell Culture-Adapted Sudan Virus or Ebola Virus

**DOI:** 10.3390/v10110642

**Published:** 2018-11-16

**Authors:** Kendra J. Alfson, Laura E. Avena, Michael W. Beadles, Gabriella Worwa, Melanie Amen, Jean L. Patterson, Ricardo Carrion, Anthony Griffiths

**Affiliations:** 1 Department of Virology and Immunology, Texas Biomedical Research Institute, San Antonio, TX 78227, USA; kalfson@txbiomed.org (K.J.A.); lavena@bu.edu (L.E.A.); mbeadles41@gmail.com (M.W.B.); gabriella.worwa@nih.gov (G.W.); melanieamen@yahoo.com (M.A.); jpatters@txbiomed.org (J.L.P.); rcarrion@txbiomed.org (R.C.J.); 2 Current Affiliation: Graduate School of Biomedical Sciences, University of Texas Health Science Center at San Antonio, San Antonio, TX 78229, USA; 3 Current Affiliation: Institute of Environmental Health, 9330 Corporate Drive, Selma, TX 78154, USA; 4 Current Affiliation: National Institute of Allergy and Infectious Diseases (NIAID), Integrated Research Facility, Division of Clinical Research, 8200 Research Plaza, Fort Detrick, MD 21702, USA; 5 Current Affiliation: Department of Microbiology and National Emerging Infectious Diseases Laboratories, Boston University School of Medicine, 620 Albany St, Boston, MA 02118, USA

**Keywords:** Ebola virus, Sudan virus, nonhuman primate model, low dose

## Abstract

The filoviruses Ebola virus (EBOV) and Sudan virus (SUDV) can cause severe diseases, and there are currently no licensed countermeasures available for use against them. Transmission occurs frequently via contact with bodily fluids from infected individuals. However, it can be difficult to determine when or how someone became infected, or the quantity of infectious virus to which they were exposed. Evidence suggests the infectious dose is low, but the majority of published studies use high exposure doses. This study characterized the outcome of exposure to a low dose of EBOV or SUDV, using a *Macaca fascicularis* model. Further, because the effect of virus passage in cell culture may be more pronounced when lower exposure doses are used, viruses that possessed either the characteristics of wild type viruses (possessing predominantly 7-uridine (7U) genotype and a high particle-to-plaque forming unit (PFU) ratio) or cell culture-passaged viruses (predominantly 8-uridine (8U) genotype, a lower particle-to-PFU ratio) were used. The time to death after a low dose exposure was delayed in comparison to higher exposure doses. These data demonstrated that an extremely low dose of EBOV or SUDV is sufficient to cause lethal disease. A low dose exposure model can help inform studies on pathogenesis, transmission, and optimization of prevention strategies.

## 1. Introduction

The filoviruses Ebola virus (EBOV) and Sudan virus (SUDV) are enveloped, negative-sense, single-stranded, RNA viruses that can cause disease with high case fatality rates [1], and there are currently no licensed countermeasures available for use against them. Since being discovered in 1976, EBOV and SUDV have caused intermittent outbreaks, primarily in the Democratic republic of Congo (EBOV) or in Uganda and South Sudan (SUDV) [2]. In 2014, EBOV caused the largest recorded filovirus outbreak in western Africa [3].

In many of the initial outbreaks, contaminated needles were implicated in transmission [4]. Now, direct contact with infected individuals and their bodily fluids is thought to be the primary transmission route [5]. However, in large outbreaks with sustained person-to-person transmission, it is often difficult to determine when or how an individual became infected, and it is almost impossible to determine what quantity of infectious virus a person was exposed to [6]. Furthermore, when patients are not acutely ill, viral loads can be low. These low loads are thought to be unimportant epidemiologically [5,7], but further study is needed to fully understand the implications of low dose exposures. Moreover, a better understanding of the outcomes of exposure to a low dose is needed, given the evidence that filoviruses can persist within previously infected individuals for months [8]. It remains unclear how persistent infection occurs, or whether persistently infected individuals pose a threat of infection to others [8,9].

During animal exposure studies, the effect of using virus that has been passaged in cell culture may be more pronounced when lower exposure doses are used. For example, EBOV and SUDV can generate multiple products from the glycoprotein (*GP*) gene via RNA editing. At the *GP* RNA editing site, the wild type virus genome contains seven uridines (7U) and without editing, messenger RNA transcripts contain seven adenosines and produce soluble GP, a truncated form of full length GP. When editing occurs, an eighth adenosine is incorporated into the transcript and full length GP is produced [10]. Cell culture passaging of EBOV and SUDV can cause the genomic sequence at this editing site to expand to eight uridines (8U) [11,12,13]. Different forms of GP are thought to play a role in pathogenicity, but the various functions and their importance are not fully understood. Crucially, the influence of changes at this locus in vivo remain unclear. The editing site genotype does not appear to affect survival. There does not appear to be a difference in survival when animals are exposed to a virus stock with a predominantly 7U genotype (7U virus stock) versus a virus stock with a predominantly 8U genotype (8U virus stock) EBOV or SUDV, but disease course may be impacted [13,14]. Furthermore, another characteristic of cell culture-passaged viruses is altered particle-to-plaque forming unit (PFU) ratio; filovirus particle-to-PFU ratios have been shown to decrease after cell culture passage [15,16]. Previous studies have also shown that using a lower exposure dose can help to uncover differences between different virus stocks [15,16].

It is known that filoviruses are highly potent, and that only a small number of infectious particles are needed to cause death [15,17,18,19]. Yet, the majority of published studies use a high exposure dose of 100 or 1000 PFU; very few studies have investigated the consequences of exposure with fewer than 10 PFU. This study was designed to characterize the outcome of exposure to a low dose of EBOV or SUDV, using the *Macaca fascicularis* model of infection. A low dose exposure model can help to inform studies on pathogenesis, public health countermeasure development, and the optimization of transmission prevention strategies.

## 2. Methods

### 2.1. Ethics Statement

Animal research was conducted under a Texas Biomedical Research Institute Institutional Animal Care and Use Committee-approved protocol (protocol 1381, 20 August 2013) in compliance with the Animal Welfare Act and other federal statutes and regulations relating to animals and experiments involving animals. The facility where this research was conducted is accredited by the Association for Assessment and Accreditation of Laboratory Animal Care International and adheres to principles stated in the 8th Edition of the Guide for the Care and Use of Laboratory Animals, National Research Council [20]. Nonhuman primates (NHPs) were single housed, fed commercially available monkey biscuits (Purina, Gray Summit, MO, USA), and provided with commercial toys and dietary enrichment. During blood collections, nonhuman primates (NHPs) were anesthetized using tiletamine HCl and zolazepam HCl (Zoetis Inc., Parsippany-Troy Hills, NJ, USA), and euthanasia was performed using an intravenous overdose of sodium pentobarbital (Vortech Pharmaceuticals, Dearborn, MI, USA).

### 2.2. Clinical Scoring System and Euthanasia Criteria

Nonhuman primates were observed at least twice daily for up to 21 days post-exposure, at which time any survivors were euthanized. Clinical scores were reported to the responsible veterinarian, and euthanasia was approved when scores indicated that an NHP was terminally ill. A brief description of the clinical scoring system (described previously in [16]) is as follows. Animals received one point each for reductions in feed or fluid intake, dehydration, no stool production, rough hair coat, nasal discharge, or bleeding at the blood collection site. Animals received two points each for reduced stool production, not drinking, or bleeding from somewhere other than a blood collection site. Rectal temperature and petechia were scored on a scale of one to three, and body weight reductions were scored as one or two, depending on severity. Responsiveness was also scored on a scale: slightly diminished general activity (one point), reduced response to external stimuli (two points), moderate to dramatically reduced response to external stimuli (eight points), or severely unresponsive (fifteen points, immediate euthanasia). Animals received three points for labored breathing or eight points for agonal breathing. Euthanasia criteria were developed to minimize undue pain and distress, and included a combination of severe petechia or bleeding, complete anorexia for 24 hours, a temperature change of greater than five degrees (°F) from baseline, moderate to dramatically reduced response to external stimuli, respiratory distress, thrombocytopenia, or severe elevation of gamma-glutamyl transferase (GGT), alanine aminotransferase (ALT), alkaline phosphatase (ALP), or blood urea nitrogen (BUN).

### 2.3. Cells and Virus

BEI-sourced Vero E6 cells (VERO C1008, catalog number NR-596, BEI resources; African green monkey kidney origin) were grown at 37 °C with five percent CO_2_ in normal growth media that consisted of minimum Essential Media (Gibco, Grand Island, NY, USA) containing 2 mM l-glutamine (Gibco, Grand Island, NY, USA) and 1 mM sodium pyruvate (Gibco, Grand Island, NY, USA) with 10% heat-inactivated fetal calf serum (FCS, Gibco, Grand Island, NY, USA). Starting virus material consisted of *Zaire ebolavirus* isolate Ebola virus/*H. sapiens*-tc/COD/1995/Kikwit-199510621 and *Sudan ebolavirus* isolate Sudan virus/*H. sapiens*-tc/SDN/2000/Gulu-200011676, both passage number 2 (P2) on Vero E6 cells, acquired from Dr. T. Ksiazek, University of Texas Medical Branch. The 7U virus material used for NHP exposures was generated by amplifying P2 virus once in Vero E6 cells at a multiplicity of infection (MOI) of 0.001. The 7U EBOV stock was generated in September 2012 (lot number 201209171), and the abundance of genomes with 7U at the editing site locus was approximately 94%. The 7U SUDV stock was generated in June 2012 (lot number 201206211), and the abundance of genomes with 7U at the editing site locus was approximately 75%. The 8U virus used for SUDV exposure was generated (April 2013) by amplifying the aforementioned P3 virus 10 additional times [13]; the abundance of genomes with 8U at the editing site locus was approximately 75%. The 8U EBOV was obtained from U.S. Army Medical Research Institute of Infectious Diseases and then passaged once in Vero E6 cells at a low MOI (August 2010, lot number 201008111) and has been reported previously [15]; the abundance of genomes with 8U at the editing site locus was approximately 94%. All stocks were stored in liquid nitrogen after generation.

### 2.4. Determination of Viral Particle Concentration

Transmission electron microscopy, using a JEOL 100CX microscope (JEOL USA, Inc., Peabody, MA 01960, USA), was used to determine the particle concentration of each virus stock, as previously described [13,15]. The calculated number of viral particles per mL was divided by the number of PFU per mL to yield particle-to-PFU ratios. The viral particle concentration for all four virus exposure stocks was determined in August 2013, fewer than six months prior to the animal exposures.

### 2.5. Determination of Viral Titers

Viral titers were determined by plaque assay using either a methylcellulose and crystal violet assay, or an agarose and neutral red assay (previously described in [21]). The methylcellulose and crystal violet assay was used to determine the titer of the NHP exposure dose for the 8U EBOV dose study (as previously described in [15]), while the agarose and neutral red assay was used for all other viral titrations. As expected, all of the virus exposure doses were below the assay limit of detection, consistent with previous work showing that some particles do not yield plaques in cell culture, but that they can nonetheless cause lethal disease in NHPs [15,16]. When the exposure doses were prepared, calculations were performed using viral titers determined previously (approximately one year prior to the animal exposures for all stocks except the 8U EBOV). However, back titer plaque assays were also performed on the day of animal exposure to verify the viral titer.

### 2.6. Deep Sequencing and Sample Preparation

RNA was extracted from serum collected at time of death from all NHPs, and subjected to deep sequencing, as described before [13,22]. Briefly, the serum was diluted in TRIzol LS reagent (Invitrogen, Grand Island, NY, USA), and RNA was extracted following the manufacturer’s instructions. Clean up steps were performed (DNA, rRNA, and mRNA were removed) to increase the sequence depth of the viral material. An Illumina TruSeq total RNA sample preparation kit (Illumina, Inc., San Diego, CA, USA) was used to prepare RNA libraries for sequencing, according to the manufacturer’s instructions. An Illumina pipeline was used to generate FASTQ files to begin data analysis. Files containing all sequence reads were mapped to the reference sequence (GenBank accession numbers: AY729654 (SUDV) and AY354458 (EBOV) using Lasergene SeqMan NGen (DNASTAR, Madison, WI, USA). The depth of nucleotide coverage was determined at each position in the genome.

### 2.7. Experimental Inoculation of Macaca fascicularis

Sixteen male *Macaca fascicularis* were intramuscularly (IM) injected in the deltoid muscle with a target dose of 0.01 PFU of a 7U virus stock (*n* = 4) or 8U virus stock (*n* = 4) of SUDV, or a 7U virus stock (*n* = 4) or 8U virus stock (*n* = 4) of EBOV (previously described in [15]). Nonhuman primates (Vietnamese origin), 3 to 4.5 years of age and 2.6 to 4.1 kg in weight, were acquired from Covance, and serum tested to ensure no reactivity to the filovirus antigen prior to purchase. Nonhuman primates were shipped directly to Texas Biomedical Research Institute for a standard quarantine period. This quarantine period allowed for the NHPs’ health status to be evaluated, and for the NHPs to acclimate to their caging and diet. Virus exposure occurred on study day zero. Blood samples were collected on days 0, 3, 5, 7, 10, 14, and 21 post-exposure, for analyses of serology, hematology, clinical chemistry, coagulation parameters, and viral load determination. During each scheduled blood collection, the rectal temperature was taken, and the weight was recorded. When moribund, or at 21 days post-exposure, the NHPs were euthanized and necropsy was performed, and gross pathologic findings were noted.

### 2.8. Hematology, Coagulation, and Blood Chemistry

Biochemical analysis was performed using the mammalian liver enzyme profile rotor on a Vet Scan analyzer (Abaxis, Inc., Union City, CA, USA). Complete blood counts were performed using a Vet HM2 machine (Abaxis, Inc.). Coagulation times were determined using the IDEXX Coag Dx Analyzer (IDEXX laboratories, Westbrook, ME, USA). Clinical Pathology at Texas Biomed has determined normal ranges for cynomolgus macaques housed under laboratory conditions at Texas Biomed facilities.

### 2.9. Statistics

The log-rank Mantel–Cox test was used to analyze the survival curves.

## 3. Results

### 3.1. Survival

To determine the outcome of low dose filovirus exposure in NHPs, sixteen *M. fascicularis* were exposed intramuscularly (IM) with a target dose of 0.01 PFU of either EBOV (*n *= 8) or SUDV (*n *= 8). Two different virus stocks were used for both EBOV and SUDV exposures: a 7U virus stock (high particle to PFU ratio, characteristic of wild type virus), or an 8U virus stock (characteristic of cell culture passaged virus, lower particle to PFU ratio). Within each virus exposure group, four animals were exposed to the 7U virus stock, and the other four were exposed to the 8U virus stock (Table 1).

**Figure 1:**
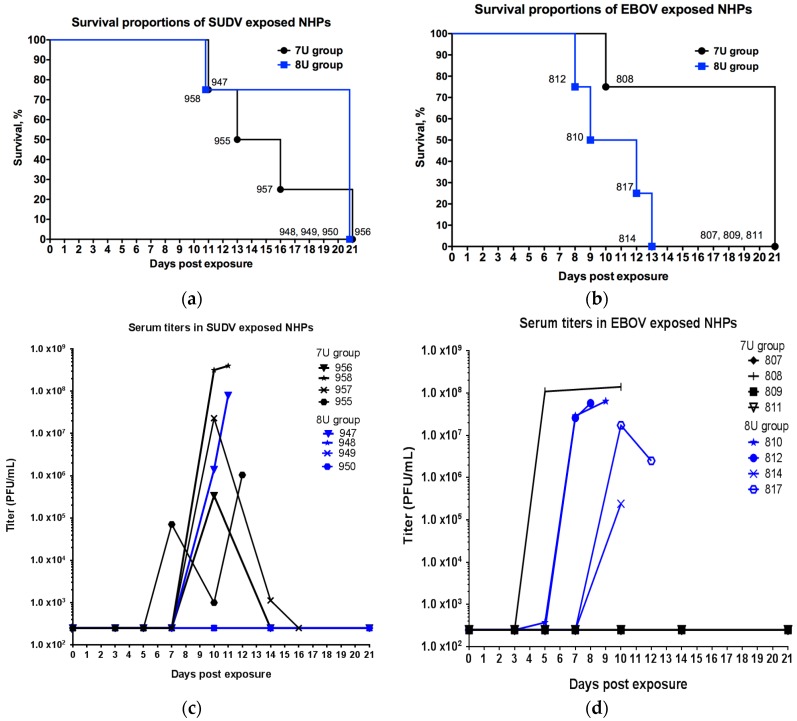
Survival and viremia in *M. fascicularis* experimentally exposed to 0.01 plaque-forming units of either Sudan virus (SUDV) or Ebola virus (EBOV) with 7-uridine (7U) or 8-uridine (8U) genotype. NHPs—nonhuman primates. Numbers in the figure refer to individual animal identification numbers. (**a**) Survival proportions of SUDV exposed NHPs. (**b**) Survival proportions of EBOV-exposed NHPs (8U exposed animals described previously in [15]). (**c**) Serum titers in SUDV-exposed NHPs. (**d**) Serum titers in EBOV-exposed NHPs (8U-exposed animals described previously in [15]).

The time to death for all animals is summarized in Table 2 and Figure 1. After exposure to 0.01 PFU of SUDV, one out of four NHPs exposed to the 7U virus stock and three out of four NHPs exposed to the 8U virus stock survived. The median time to death was 14.5 days for the 7U group and undefined for the 8U group; there was no significant difference in the time to death between the two groups (*p* = 0.2511, log-rank Mantel–Cox test). After exposure to 0.01 PFU of EBOV, three out of four NHPs exposed to 7U virus stock and zero out of four NHPs exposed to the 8U virus stock survived. The median time to death was undefined for the 7U group, and 10.5 days for the 8U group; there was no significant difference (*p* = 0.0510, log-rank Mantel–Cox test).

**Figure 2:**
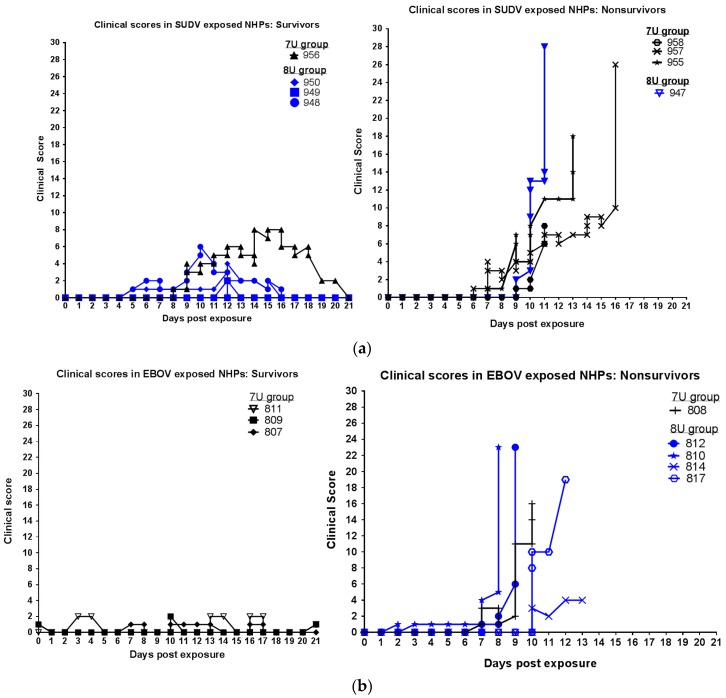
Clinical scores in *M. fascicularis* experimentally exposed to 0.01 plaque-forming units of either Sudan virus (SUDV) or Ebola virus (EBOV) with a 7-uridine (7U) or 8-uridine (8U) genotype. NHPs—nonhuman primates. (**a**) Clinical scores of SUDV-exposed NHPs. (**b**) Clinical scores of EBOV-exposed NHPs (8U exposed animals described previously in [15]).

Animals were observed at least twice daily for up to 21 days post-exposure, at which time, any survivors were euthanized. Clinical scores over the course of the study for all animals are displayed in Figure 2. Table 2 shows the final score for each animal at the time of death, and shows the manner of death (e.g., euthanasia versus found dead). For SUDV-exposed animals that were euthanized due to moribundity, at the time of euthanasia, all were scoring for: reduced responsiveness (diminished activity, withdrawn, or prostrate in cage); rough hair coat; combinations of reduced feed, enrichment, and/or fluid intake (often accompanied by dehydration); reduced stool output; two also exhibited petechia and bleeding, and two exhibited temperature changes greater than or equal to 3 °F. For EBOV-exposed animals that were euthanized due to moribundity, at the time of euthanasia all were scoring for: reduced responsiveness (diminished activity, withdrawn, or prostrate in cage); temperature change (greater than or equal to 2 °F change); petechia; combinations of reduced feed, enrichment, and/or fluid intake (often accompanied by dehydration); reduced stool output; three of four also exhibited bleeding, rough hair coat, and labored breathing.

### 3.2. Viremia

On each scheduled sedation day (days 0, 3, 5, 7, 10, 14 post-exposure), and when possible at the time of death, blood was collected from all NHPs for determination of viremia (Figure 1). For the SUDV-exposed NHPs, no animals had detectable levels of infectious virus in the serum collected on days 3 or 5 post-exposure. For NHPs that did not survive SUDV exposure (*n* = 4), three had titers greater than 1 × 10^7^ PFU/mL at the time of death, and animal 956, which survived its exposure to 7U virus stock, had a serum titer of 3.4 × 10^5^ PFU/mL on day 10 post-exposure. All NHPs that survived exposure to the 8U stock of SUDV (*n* = 3) exhibited no detectable levels of infectious virus throughout the study. For EBOV-exposed NHPs, in the 7U exposure group, only the NHP that did not survive exhibited evidence of viremia. All NHPs exposed to the 8U virus stock of EBOV had high serum titers on or near the day of death [15].

Most NHPs that survived exposure (EBOV-exposed animals 807, 809, and 811; SUDV-exposed animals 948, 949, and 950) exhibited no detectable levels of infectious virus throughout the study. Thus, it was possible the low infective dose did not result in infection. Deep sequencing was used in an attempt to determine the likelihood of infection. RNA was extracted from the serum collected at the time of death (or the closest available time point; animal 814 was found dead on day 13 post-exposure, animal 958 was found dead on day 11 post-exposure, and animal 947 expired on day 11 post-exposure before blood could be collected, so that RNA was extracted from the sample from day 10 post-exposure), and subjected to deep sequencing. Sequence reads were mapped to EBOV or SUDV genomes to determine whether there was evidence of viral RNA; the number of total sequence reads and median depth across the genome were calculated.

Values for all EBOV-exposed animals were generally high. On average, animals that did not survive exposure to EBOV virus exhibited 1,059,602 sequence reads mapped to the genome (a range of 119,048 to 2,977,753), while animals that did survive exposure to EBOV virus exhibited an average of 19,131 sequence reads mapped to the genome (a range of 6111 to 28,222). Overall, (individual animal data displayed in Table 3), animals that did not survive exposure to SUDV virus exhibited 530,309 sequence reads mapped to the genome, while animals that did survive exposure to SUDV virus exhibited 5193 sequence reads mapped to the genome.

### 3.3. Clinical Pathology

Rectal temperatures were taken on each scheduled sedation day (days 0, 3, 5, 7, 10, 14 post-exposure) and when possible, at the time of death. For SUDV-exposed animals, the 7U group exhibited higher temperatures than the 8U group. The 7U stock of SUDV-exposed animals exhibited febrility between days 7 and 14 post-exposure, temperatures of 39.7 to 40.2 °C (1.3 to 2 °C increases when compared to day 0 post-exposure). Only one NHP in the 8U SUDV group (the nonsurviving animal) exhibited febrility, a temperature of 39.6 °C (a 1.7 °C increase when compared to day 0) on day 10 post exposure. For EBOV-exposed animals, the 7U group did not exhibit substantial increases and the 8U group exhibited rectal temperatures of 39.4 to 39.9 °C (increases of 1 to 2.6 °C when compared to day 0), shortly before death (as described previously [15]).

**Figure 3:**
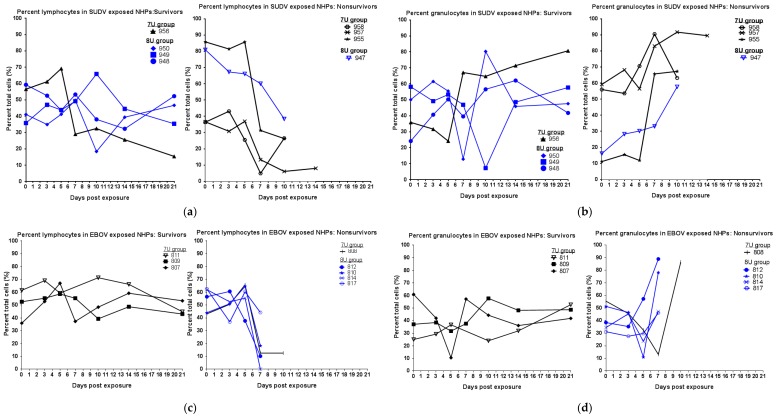
Lymphocyte and granulocyte percentages in *Macaca fascicularis* exposed to low doses of Sudan virus (SUDV) or Ebola virus (EBOV). *M. fascicularis* were experimentally exposed to 0.01 plaque-forming units of either SUDV or EBOV with 7-uridine (7U) or 8-uridine (8U) genotype. During each scheduled sedation, and when possible at the time of death, blood specimens were collected and analyzed. Results of hematology analysis for all animals throughout the course of the study are shown. Normal values determined at the Texas Biomed clinical pathology lab—Lymphocytes: 43–77% and Granulocytes: 19–52%. NHPs—nonhuman primates. (**a**) Percentage of lymphocytes in SUDV-exposed NHPs. (**b**) Percentage of granulocytes in SUDV-exposed NHPs. (**c**) Percentage of lymphocytes in EBOV-exposed NHPs. No data are available for animal 811 on day 7 post-exposure. (**d**) Percentage of granulocytes in EBOV-exposed NHPs. No data are available for animal 811 on day 7 post-exposure.

**Figure 4:**
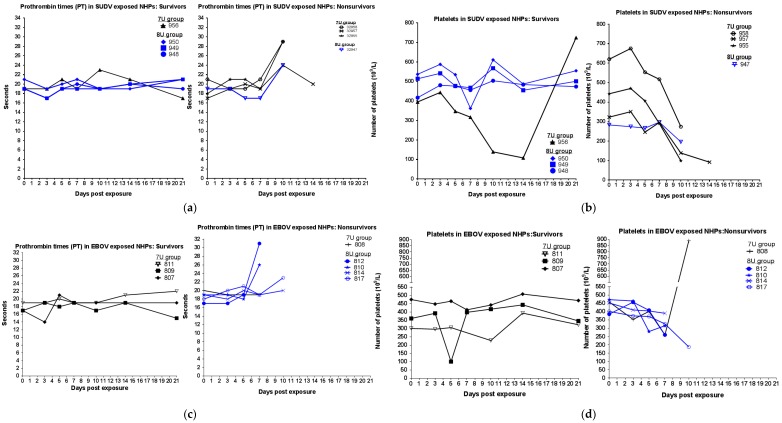
Coagulation parameters in *Macaca fascicularis* exposed to low doses of Sudan virus (SUDV) or Ebola virus (EBOV). *M. fascicularis* were experimentally exposed to 0.01 plaque forming units of either SUDV or EBOV with 7-uridine (7U) or 8-uridine (8U) genotype. During each scheduled sedation, and when possible at the time of death, blood specimens were collected and analyzed. Results of coagulation analysis for all animals throughout the course of the study are shown. NHPs—nonhuman primates. (**a**) Prothrombin times (PT) in SUDV-exposed NHPs. (**b**) Platelets in SUDV-exposed NHPs. (**c**) Prothrombin times (PT) in EBOV-exposed NHPs. (**d**) Platelets in EBOV-exposed NHPs.

**Figure 5:**
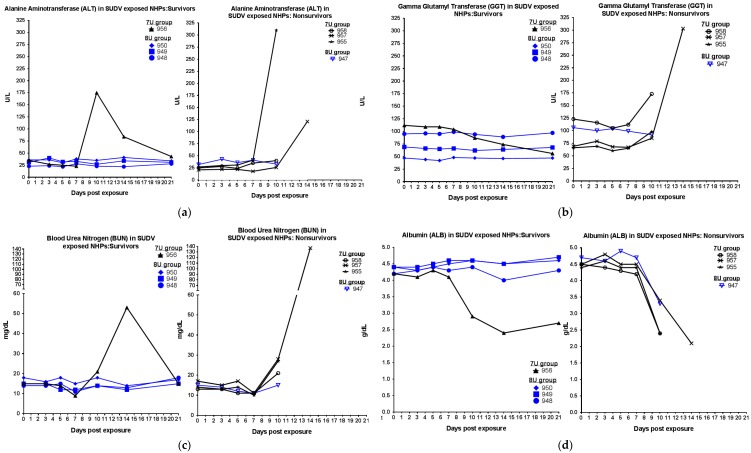
Clinical chemistry parameters in *Macaca fascicularis* exposed to low doses of Sudan virus (SUDV). *M. fascicularis* were experimentally exposed to 0.01 plaque-forming units of SUDV with either 7-uridine (7U) or 8-uridine (8U) genotypes. During each scheduled sedation, and when possible at the time of death, blood specimens were collected and analyzed. Results of clinical chemistry analysis for all SUDV-exposed animals throughout the course of the study are shown. NHPs—nonhuman primates. (**a**) Alanine aminotransferase (ALT) in SUDV-exposed NHPs. (**b**) γ glutamyl transferase (GGT) in SUDV-exposed NHPs. (**c**) Blood urea nitrogen (BUN) in SUDV-exposed NHPs. (**d**) Albumin (ALB) in SUDV-exposed NHPs.

During scheduled sedations, blood samples were taken for the analysis of blood chemistry, coagulation times, and hematology. For SUDV-exposed NHPs, hematological analysis showed that over the duration of the study, in all animals, the percentage of granulocytes increased, while the percentage of lymphocytes decreased (Figure 3), consistent with SUDV virus exposure in NHPs [13]. Coagulation abnormalities were observed in some animals that were exposed to the 7U SUDV stock (Figure 4); prothrombin times were above the normal range for two of four NHPs, and platelet levels were decreased in four of four NHPs, including the surviving animal. Conversely, within the 8U SUDV exposure group, PT times did not fall outside the normal range, and platelet levels did not exhibit sustained decreases. The difference in PT times between the 7U and 8U exposure groups was most apparent on day 10 post-exposure. Additionally, abnormal blood chemistry values were primarily observed within the 7U SUDV exposure group (Figure 5). Only animals in the 7U group exhibited increased ALT, GGT, and BUN values. The survivor from this group exhibited elevated ALT values (five times greater than baseline on day 10 post-exposure) and BUN values (3.5 times greater than baseline on day 14 post-exposure). All 7U SUDV-exposed NHPs also exhibited decreased ALB values after day 7 post-exposure; within the 8U exposure group, only the NHP that did not survive exhibited decreased ALB. As with PT times, the difference in blood chemistry values between the 7U and 8U exposure groups was most apparent on day 10 post-exposure.

**Figure 6:**
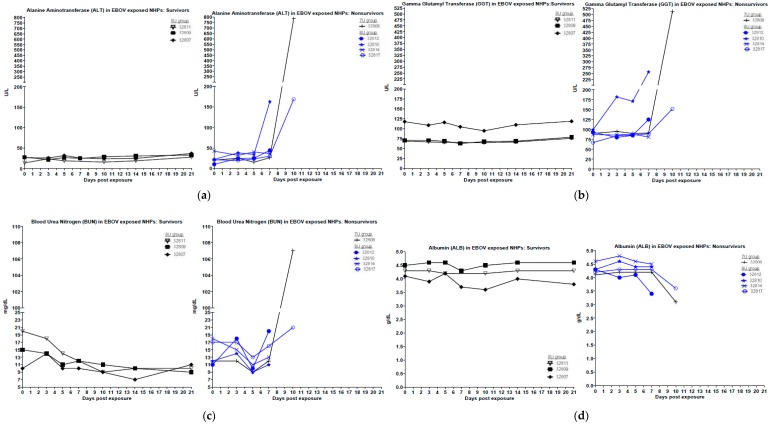
Clinical chemistry parameters in *Macaca fascicularis* exposed to low doses of Ebola virus (EBOV). *M. fascicularis* were experimentally exposed to 0.01 plaque forming units of EBOV with either 7-uridine or 8-uridine genotypes. During each scheduled sedation, and when possible at the time of death, blood specimens were collected and analyzed. Results of clinical chemistry analysis for all EBOV-exposed animals throughout the course of the study are shown. No data are available for animal 32811 on day 7 post-exposure. NHPs—nonhuman primates. (**a**) Alanine aminotransferase (ALT) in EBOV-exposed NHPs. (**b**) γ glutamyl transferase (GGT) in EBOV-exposed NHPs. (**c**) Blood urea nitrogen (BUN) in EBOV-exposed NHPs. (**d**) Albumin (ALB) in EBOV-exposed NHPs.

For EBOV-exposed animals, the NHPs that did not survive exhibited increased granulocytes and decreased lymphocytes after days 3 to 5 post-exposure (Figure 3). Two out of three survivors exposed to 7U stocks of EBOV also exhibited similar changes after day 5 post-exposure, but the values rebounded to baseline by project end. None of the NHPs exposed to a 7U stock of EBOV exhibited prolonged PT times, and only the NHP that succumbed to exposure exhibited decreased platelets (day 5 post-exposure) (Figure 4). Three out of four NHPs exposed to 8U EBOV exhibited prolonged PT times and decreased platelets at or near the time of death. The difference in PT times between the 7U and 8U exposure groups was most pronounced on day 7 post-exposure. Figure 6 shows blood chemistry values for the 7U and 8U EBOV exposure groups. The NHP that did not survive exposure to the 7U stock of EBOV exhibited increased ALT, GGT, and BUN values, along with decreased ALB at or near the time of death. None of the other NHPs exposed to the 7U stock of EBOV exhibited similar abnormalities. Two out of four 8U EBOV-exposed NHPs exhibited increased ALT and GGT values at or near the time of death. Albumin was also decreased, and BUN increased in two out of four 8U stocks of EBOV-exposed animals.

### 3.4. Anatomical Pathology

There were no clear differences in anatomical pathology between the 7U and 8U exposure groups for both SUDV and EBOV, and findings were similar to what would be expected from exposure with a higher dose. Animals that did not survive exposure exhibited similar macroscopic and microscopic findings, consistent with acute filovirus infection (Table 4). Common gross abnormalities included petechia, splenic or hepatic pallor, firm or dark lymph nodes, and testicular hemorrhage or redness. Common histologic lesions consistent with acute filovirus infection, included: splenic lymphoid depletion with necrosis, hemorrhage, and fibrin, lymph node lymphoid depletion and/or necrosis, and hepatocellular necrosis (Table 4). Survivors did not exhibit macroscopic or microscopic abnormalities.

## 4. Discussion

Herein, we characterized the outcome of exposure to a low dose of SUDV and EBOV, in the *M. fascicularis* model of infection. *M. fascicularis* were exposed to 0.01 PFU of either SUDV or EBOV. The virus used for exposure was either predominantly the 7U genotype (characteristics of wild type virus) or predominantly the 8U genotype (characteristics of cell culture adapted virus). We found that exposure to a target dose of 0.01 PFU of the 7U stock of SUDV was lethal in 75% of NHPs, while the 8U stock caused lethality in only 25% of NHPs. Viremia and clinical pathology data indicated that 100% of the NHPs exposed to the 7U stock of SUDV became infected. One animal succumbed to exposure to the 8U stock of SUDV, while the three NHPs that survived exposure showed some evidence of possible infection via deep sequencing, which yielded reads mapped to the SUDV genome. Conversely, exposure to a target dose of 0.01 PFU of the 7U stock of EBOV caused lethality in only 25% of NHPs, while previously published data show that 0.01 PFU of an 8U stock is 100% lethal [15]. Sequencing data (number of reads mapped to EBOV genome) indicated that 100% of the NHPs exposed to the 8U stock became infected, one animal succumbed to exposure to the 7U stock, and the three NHPs that survived exposure to the 7U stock EBOV showed evidence of viral RNA in the serum, suggestive of infection.

In previous studies, subtle differences were found between EBOV stock of viruses with predominately 7U and 8U genotypes when tested in animals (e.g., delayed disease course, delayed febrility, delayed serum chemistry changes) [14] but the SUDV pathogenesis appeared to be even less impacted by the editing site genotype [13]. In the present study, while differences in survival were not statistically different for either virus, this could be related to the size of the study, and an impact of genotype on survival cannot be discounted. There were also temporal differences in the disease course. Abnormalities in hematology, coagulation, and blood chemistry were primarily observed within the 7U SUDV and 8U EBOV exposure groups. Over the duration of the study, in all SUDV-exposed animals and all nonsurvivor EBOV-exposed animals, the percentage of granulocytes increased, while percentage of lymphocytes decreased. Coagulation abnormalities (elevated PT times and decreased platelets) were observed for some of the 7U SUDV and 8U EBOV groups. Conversely, within the 8U SUDV and 7U EBOV exposure groups, PT times were not elevated, and only the NHP that succumbed to the 7U stock of EBOV exposure exhibited decreased platelets. Additionally, abnormal blood chemistry values (increased ALT, GGT, or BUN values, and decreased ALB values) were primarily observed within the 7U SUDV and 8U EBOV exposure groups. The NHP that succumbed within the 8U SUDV exposure group exhibited decreased ALB only, and only the NHP that succumbed within 7U EBOV exposure group exhibited increased ALT, GGT, and BUN values, along with decreased ALB near the time of death.

It is important to note though, the difference in survival between the 7U SUDV and 8U SUDV groups could also be related to the different particle to PFU ratios of the two SUDV stocks used. Filovirus particle to PFU ratios have been shown to decrease during cell culture adaptation [15,16], so that the 8U-exposed group received fewer particles than the 7U-exposed group. Previously, we have also considered that differences in the particle to PFU ratios may be caused by different morphologies or different degrees of virus aggregation between stocks. However, we found no evidence to support this hypothesis [15]. Thus, while it is clear that passage history of the virus used in exposure studies has an impact, it remains unclear as to whether the differences are related to the editing site genotype or to specific infectivity. Interestingly, the Makona variant of EBOV was shown not to be lethal at a low dose when the NHPs were exposed orally or conjunctivally [19]. However, this could be due to differences in particle counts, differences between the variants [23] (though of note, this Kikwit and Makona comparison study was performed in rhesus macaques), or differences between exposure routes [24].

Potential differences between the 7U and 8U SUDV exposure groups are not surprising, as the virus possessing characteristics of the wild type virus was lethal in the majority of exposed animals, while the virus possessing the characteristics of cell culture-adapted virus resulted in a lower lethality. One would expect that the cell culture passaged virus may be attenuated in vivo. However, any differences in the EBOV exposure groups are not necessarily expected, as the virus possessing characteristics of cell culture-adapted virus is the stock that appeared to be more lethal than the virus possessing the characteristics of the wild type virus. This could be related to the methods that were used to generate the stocks; the predominantly 8U genotype SUDV, possessing the characteristics of a cell culture-adapted virus, was generated through serial passage in cell culture, while the predominantly 8U genotype EBOV, possessing the characteristics of a cell culture-adapted virus, was passaged in cell culture but not to the same extent as the SUDV virus. This may also be a product of the low dose system used herein. However, due to the small group numbers, it is difficult to draw conclusions.

The time to death after low dose exposures to both SUDV and EBOV was delayed in comparison to that observed by the authors during studies performed at the same time, but using higher exposure doses [13,15]. The median time to death in similarly designed studies using a dose of 100 PFU is 8.5 to 10 days for SUDV [13], compared to 14 days in this study, and 6 to 7 days for EBOV [15,24,25], compared to 10.5 days in this study.

However, this timeframe may more closely resemble the time to death observed during human infections [26,27]. In other published studies using lower doses, a delay in time to death has also been observed [18]. Animal models designed to assess public health countermeasure efficacy should recapitulate human disease as closely as possible [28], and using a lower exposure dose may better help mimic human infections, including the time to death. Different doses can also result in different viral replication kinetics and immune responses during infection. Modeling these different scenarios is important for gaining a better understanding of pathogenesis. Thus, it will be important to determine whether the low dose that was demonstrated to be lethal in NHPs is relevant to humans.

These data also demonstrate that a low number of particles is sufficient to cause death, and they are consistent with previous work showing that some particles do not yield plaques in cell culture, but they can nonetheless cause lethal disease. Previous studies have used a range of doses, and they have demonstrated a lethal dose as low as approximately 2 PFU [18,28]. Our previous work has shown that for EBOV (predominantly 8U genotype) [15] and MARV [16], a dose of 0.01 PFU is sufficient to cause death, and that fewer than 100 particles is lethal. However, such low dose studies had not been previously performed with SUDV or predominantly 7U genotype EBOV.

More information about low dose exposures is needed to better understand transmission and persistence. Virus has been found in a variety of bodily fluids from infected individuals, including blood, semen, saliva, and sweat [29] but it is difficult to determine how much infectious virus is usually present in these fluids [6]. Furthermore, when patients are not acutely ill, such as asymptomatic individuals or mildly ill people at the beginning stages of infection, viral loads can be very low. Moreover, evidence suggests that filoviruses can persist within previously infected individuals for months [8]. The low number of SUDV sequence reads found in serum from animals surviving SUDV exposure may be the result of persistent infection, suggesting that low dose exposure studies could be a way to model persistence. It remains unclear as to how persistent infection occurs, or whether persistently infected individuals pose a threat of infection to others [8,9], but modeling persistence and exposure to persistently infected individuals is crucial. While low viral loads are thought to be unimportant epidemiologically [5,7], with increasing evidence that low doses of virus can be lethal, further study is needed to understand the potential for transmission from these individuals. Future studies should focus on more accurately quantifying the amount of infectious virus in bodily fluids and shed by asymptomatic individuals or persistently infected individuals, to better evaluate the risks posed.

Finally, studies to develop and test public health countermeasures may benefit from the use of different exposure doses. The higher doses that are frequently used may be too stringent for suitable medical countermeasure testing. For example, studies with influenza have shown that high exposure doses can have exaggerated effects that increase pathogenicity and that mask the activity of antivirals, and thus, lower doses are often used when testing such countermeasures [30].

In summary, we found that a low number of Ebola virus or Sudan virus particles is sufficient to cause death, and modeling the consequences of low dose exposure is important for gaining a better understanding of pathogenesis, the prevention of transmission, and countermeasure developments.

## Figures and Tables

**Table 1:** Virus exposure stocks.

Virus	Genotype	Particles per PFU	Calculated no. Particles in Target Dose	Cohort Size	Survivors
SUDV	7U (75%)	6 × 10^4^	600	4	1/4
	8U (75%)	3 × 10^3^	30	4	3/4
EBOV	7U (94%)	2 × 10^4^	200	4	3/4
	8U (94%) [15]	8 × 10^3^	80	4	0/4

**Table 2:** Mortality Information.

Animal ID	Exposure Virus	Day of Death (Days Post-Exposure)	Manner of Death	Total Clinical Score at Death
955	SUDV, 7U	13	Euthanized	18
956	SUDV, 7U	21	Euthanized (S)	0
957	SUDV, 7U	16	Euthanized	26
958	SUDV, 7U	11	FDIC	8 *
947	SUDV, 8U	11	Expired ^1^	28
948	SUDV, 8U	21	Euthanized (S)	0
949	SUDV, 8U	21	Euthanized (S)	0
950	SUDV, 8U	21	Euthanized (S)	0
807	EBOV, 7U	21	Euthanized (S)	0
808	EBOV, 7U	10	Euthanized	16
809	EBOV, 7U	21	Euthanized (S)	0
811	EBOV, 7U	21	Euthanized (S)	0
810	EBOV, 8U	9	Euthanized	23
812	EBOV, 8U	8	Euthanized	23
814	EBOV, 8U	13	FDIC	4 *
817	EBOV, 8U	12	Euthanized	19

* Found dead in cage (FDIC); (S)—survivor, animal euthanized on final day of project; ^1^ Animal expired while veterinary technicians were preparing to euthanize.

**Table 3:** Deep sequencing of RNA extracted from the serum at (or near*) time of death: Sequence reads mapped to the SUDV or EBOV genomes.

Animal ID	Exposure Virus	Day of Death (Days Post Exposure)	Total Sequence Reads Mapped to Genome	Median Depth of Coverage
955	SUDV, 7U	13	19,095	31
956	SUDV, 7U	21	20,331	107
957	SUDV, 7U	16	7197	45
958	SUDV, 7U	11	2,053,126	40,873
947	SUDV, 8U	11	41,818	205
948	SUDV, 8U	21	195	2
949	SUDV, 8U	21	222	2
950	SUDV, 8U	21	23	2
807	EBOV, 7U	21	23,061	104
808	EBOV, 7U	10	1,819,700	37,967
809	EBOV, 7U	21	28,222	152
811	EBOV, 7U	21	6111	29
810	EBOV, 8U	9	2,977,753	29,536
812	EBOV, 8U	8	119,048	186
814	EBOV, 8U	13	254,831	3267
817	EBOV, 8U	12	126,678	919

* For animals 814, 958, and 947, RNA was extracted from the sample from day 10 post-exposure.

**Table 4:** Macroscopic and microscopic pathology findings in NHPs that succumbed to low-dose filovirus exposure (number exhibiting finding/total number of animals in group that succumbed).

Pathology Findings	SUDV 7U	SUDV 8U	EBOV 7U	EBOV 8U
Macroscopic gross pathology				
Petechia	2/3	1/1	1/1	4/4
Spleen pale	1/3	0/1	0/1	0/4
Liver pale	1/3	0/1	1/1	2/4
Lymph nodes firm, or dark	0/3	1/1	0/1	1/4
Testes red/hemorrhage	1/3	1/1	0/1	2/4
Injection site abnormality	1/3	1/1	n.d.	n.d.
Microscopic histopathology				
Splenic fibrin deposition	3/3	1/1	1/1	4/4
Splenic lymphoid depletion	3/3	1/1	1/1	4/4
Splenic necrosis	3/3	1/1	1/1	4/4
Splenic hemorrhage	2/3	1/1	1/1	3/4
Lymphoid depletion in lymph nodes	3/3	0/1	1/1	3/4
Lymph node necrosis	3/3	1/1	1/1	4/4
Adrenal gland necrosis	1/3	1/1	1/1	1/4
Testicular hemorrhage	1/3	1/1	1/1	3/4
Hepatocellular necrosis	2/3	1/1	1/1	2/4

n.d.—no data available.
